# Consensus and controversy in the management of paediatric and adult patients with ovarian immature teratoma: the Malignant Germ Cell International Consortium perspective

**DOI:** 10.1016/j.eclinm.2024.102453

**Published:** 2024-02-06

**Authors:** Farzana Pashankar, Matthew J. Murray, Joanna Gell, Nicola MacDonald, Jonathan Shamash, Deborah F. Billmire, Lindsay Klosterkemper, Thomas Olson, Michelle S. Hirsch, Michelle Lockley, Sara Stoneham, A. Lindsay Frazier

**Affiliations:** a Department of Pediatrics, Yale University School of Medicine, New Haven, CT, USA; b Department of Pathology, Tennis Court Road, University of Cambridge, CB2 1QP, UK; c Department of Paediatric Haematology and Oncology, Cambridge University Hospitals NHS Foundation Trust, Hills Road, Cambridge, CB2 0QQ, UK; d The Center for Cancer and Blood Disorders, Connecticut Children's Medical Center, Hartford, CT, USA; e Department of Pediatrics, University of Connecticut Medical School, Farmington, CT, USA; f The Jackson Laboratory for Genomic Medicine, Farmington, CT, USA; g Department of Gynaecological Surgery, University College Hospital, University College London Hospital, NHS Foundation Trust, London, UK; h Department of Medical Oncology, St Bartholomew's Hospital, London, W Smithfield, London, EC1A 7BE, UK; i Department of Surgery, Indiana University School of Medicine, Indianapolis, IN, USA; j Harvard Medical School, Boston, MA, USA; k Dana Farber/Boston Children's Cancer and Blood Center, Boston, MA, USA; l Department of Pediatrics, Aflac Cancer and Blood Disorders Center, Emory University School of Medicine, Atlanta, GA, USA; m Department of Pathology, Women's and Perinatal Division, Brigham and Women's Hospital, Harvard Medical School, Boston, MA, USA; n Department of Medical Oncology, University College Hospital, University College London Hospital, NHS Foundation Trust, London, UK; o Centre for Cancer Genomics and Computational Biology, Barts Cancer Institute, Queen Mary University of London, London, UK; p Department of Paediatrics, University College Hospital, University College London Hospital, NHS Foundation Trust, London, UK

**Keywords:** Grade, Immature teratoma, Ovarian, Stage

## Abstract

Ovarian immature teratoma (IT) is a rare neoplasm comprising ∼3% of ovarian cancers, occurring primarily in young females. Management presents several challenges, including those with elevated serum alpha-fetoprotein, potential confusion regarding pathology interpretation, and paucity of data to support decision-making. MaGIC (https://magicconsortium.com/) is an interdisciplinary international consortium of GCT experts from multiple subspecialties, with members receiving frequent queries regarding IT patient management. With evidence from published literature where available, we summarise consensus management of such patients. Given lack of published data, controversy in certain areas remains. The most obvious variance in practice is between paediatric and adult teams, despite very similar outcomes. Paediatric teams typically employ a surgery-only approach, whereas in adult practice, all patients, except those with stage IA, grade 1 (low-grade) tumours, still generally receive adjuvant chemotherapy. Given the rarity of ovarian IT and lack of published data, discussion with GCT experts and/or national advisory panels is recommended.

**Research in context**

**Evidence before this study**

Due to the rarity of ovarian immature teratoma (IT), a type of germ cell tumour (GCT), available published literature on this topic is relatively sparse. The authors in this study are members of the Malignant Germ Cell International Consortium (MaGIC; https://magicconsortium.com/); an interdisciplinary international consortium of GCT experts across multiple specialties. We sought to highlight common management queries in this condition and looked to provide recommendations based on published studies and expert opinion.

Thus, the evidence considered for this study was synthesised from each authors' clinical experience and from their literature databases/searches. The next phase involved selecting key papers, based on a ‘best evidence’ fashion involving impact and relevance, that exemplified fundamental points highlighted in the article regarding clinical management for patients with ovarian IT. This selection of representative papers was used to construct a thematically-driven, disease-based synthesis of evidence.

**Added value of this study**

We have synthesised the available literature and expert opinion from the MaGIC perspective to assist clinicians in the management of patients with ovarian IT.

**Implications of all the available evidence**

The manuscript described here represents a contemporaneous expert view for consensus clinical practice recommendations for patients with ovarian IT. This includes areas where more than one management option may be reasonable to consider, due both to current lack of consensus amongst experts and available evidence to support one approach over another.

## Introduction

Teratoma is a histological subtype of germ cell tumour (GCT) composed of tissues derived from all three embryonic layers, namely endoderm, mesoderm, and ectoderm, with varying proportions of mature and/or immature tumour elements. In children and adult females, the presence of neuroectoderm/neuroepithelium constitutes an immature teratmoma’ (IT) diagnosis. In post-pubertal males, the presence of immature versus mature elements is not distinguished nor mentioned in pathology reports. Based on the presence and proportion of immature neuroepithelial tissue present, ITs were historically graded from grade 1 to 3^1^; however, some now use a two-tier grading system which includes low-grade (previously grade 1) and high-grade (previously grade 2 and 3) tumours.^2^

IT may present in gonadal (ovarian/testicular) or extragonadal sites.^3^^,^^4^ Extragonadal sites include the sacrococcygeal region, retroperitoneum, mediastinum, head and neck; more rarely other sites such as the extremities and/or soft tissues,^3^^,^^4^ as well as intracranial location may be involved.^5^ There are clinical and epidemiological differences in patterns of presentation by site.^3^^,^^4^ Sacrococcygeal IT tumours typically are detected antentally or in the neonatal period.^3^^,^^4^ Head and neck, and intracranial IT lesions may also present antenatally or in the first few months of life, and may be life-threatening and associated with a poor prognosis.^5^ IT at all sites represent an underinvestigated patient group and more study, including molecular interrogation, is warranted, beyond the scope of this article. In this article, we focus on the management of patients with ovarian IT.

Ovarian GCT (OGCT) are rare neoplasms occurring primarily in young females between 10 and 30 years (y) of age. Ovarian IT account for 35.6% of all OGCT,^6^ but only 3% of all ovarian cancers,^7^ and thus represent an under-investigated patient group. The management of patients with ovarian IT presents several challenges, both at initial presentation and if recurrence occurs. At initial presentation, ovarian IT can present with gliomatosis peritonei (GP), which are implants of mature glial tissue that can involve the omentum, lymph nodes, or peritoneal surfaces.^8^^,^^9^ GP implants are not believed to be true metastases of IT, and instead are thought to arise from cells within the peritoneum, possibly pluripotent Mullerian stem cells.^10^^,^^11^ The presence of GP *per se* is not related to adverse outcome; however, as IT can potentially spread from the ovary to the omentum, lymph nodes, and peritoneal surfaces, all specimens need to be adequately sampled and multiple biopsies taken to exclude IT elements.^12^^,^^13^ This is important, as recurrence risks and management may alter between patients with proven evidence of GP only compared with those in whom stage III (i.e., spread beyond the ovary into the peritoneum) IT disease is confirmed.^12^^,^^13^

Another issue is the fact that ovarian IT can secrete alpha fetoprotein (AFP), detectable in the serum, making the distinction from mixed malignant GCT difficult.^3^ IT can either be ‘pure’ (100% IT) or contain microscopic foci (e.g., ≤3 mm diameter) of yolk sac tumour (YST), termed Heifetz lesions (HL).^14^ In a US study, the presence of HL correlated with higher stage, grade, and AFP levels.^14^ In completely resected tumours, presence of HL did not increase the risk of recurrence.^15^ However, the prognostic significance of HL in incompletely resected tumours remains to be determined. In contrast, a UK study showed that IT with HL were not prognostic.^3^ Further adding to the potential diagnostic confusion, IT with HL lesions are now typically reported by pathologists as mixed GCTs.^2^ Another dilemma that arises is how best to distinguish grade 3 IT from neuroepithelium that has transformed into ‘embryonic-type neuro-ectodermal tumour’ (‘ENT’),^16^ previously referred to as ‘primitive neuroectodermal tumour’ or ‘PNET’.^17^^,^^18^

If recurrence of IT occurs, there are two potential scenarios–recurrence of IT *per se*, or recurrence of IT with somatic malignant transformation (SMT), e.g., ENT. In addition, development of growing teratoma syndrome (GTS) may occur, for which three essential criteria are required. First, increase in size of the tumour mass on radiology, most typically during or shortly after the delivery of adjuvant chemotherapy (see section below); second, normalisation of previously elevated serum tumour markers (AFP); and third, presence of mature teratoma (MT) only, without any malignant component, on full histological examination of the resection specimen.^19^

As IT is a rare tumour, there is no single published consensus view on optimal patient management, either at initial presentation or at any potential recurrence. The Malignant Germ Cell International Consortium (MaGIC; https://magicconsortium.com/) is an interdisciplinary international consortium of GCT experts from paediatric, medical, and gynaecological oncology, surgery, and pathology subspecialties. As GCT experts, many of the members receive frequent questions from clinical colleagues regarding patient management of IT. Here, given the lack of published data on this subject, we summarise our consensus management approach to patients with IT. As expected, given the paucity of data, areas of controversy remain. We also highlight important research questions in this under-investigated disease. Accordingly, discussion with GCT experts and/or national advisory panels, with relevant radiological, surgical, oncological, and pathological experience, is recommended for such rare and complex patients.

## Methods

### Search strategy and collection criteria

Due to the rarity of ovarian IT, available published literature on this topic is relatively sparse. The authors in this study are MaGIC members; we sought to highlight common management queries in this condition and looked to provide recommendations based on published studies and, where published data was lacking, expert opinion. Thus, the data considered for this study was synthesised from each authors’ clinical experience and from their literature databases and searches, including MEDLINE and PubMed. Abstracts and reports from meetings were included only when relevant and no suitable full peer-reviewed articles were available. Only articles published in English between 1970 and 2023 were included.

The next phase involved selecting key papers, based on a ‘best evidence’ fashion involving impact and relevance, that exemplified fundamental points highlighted in the article regarding clinical management for patients with ovarian IT. This selection of representative papers was used to construct a thematically-driven, disease-based synthesis of evidence.

It should be noted that our study conforms to SAGER guidelines for reporting of sex and gender information.

### Role of the funding source

The authors acknowledge previous financial support for MaGIC from the St Baldrick's Foundation; grant reference number 358099. The authors thank Addenbrookes Charitable Trust (ACT; https://act4addenbrookes.org.uk/), Cambridge, UK, for providing open access funding. The funders were not involved in the study design, data collection or interpretation, nor the decision to submit for publication.

## Recommendations and discussion

### Management of IT at initial presentation

Most patients with ovarian IT present with abdominal discomfort with a palpable abdomino-pelvic mass. Some present with acute abdominal pain due to cyst or tumour rupture, haemorrhage, or ovarian torsion. Serum tumour markers (STM) such as human chorionic gonadotropin (HCG) and lactate dehydrogenase (LDH) are typically normal, however AFP can be elevated in pure IT or IT with HL. AFP is also typically elevated in mixed malignant GCTs containing foci of YST >3 mm diameter. There is no clear threshold of AFP that helps distinguish IT from such mixed GCTs, however in children treated in the UK, an arbitrary cut off of <1000 kU/L or IU/L (equivalent to <1210 ng/ml; conversion rate: 1 kU/L = 1.21 ng/ml) has been used for >25 years as a pragmatic level considered compatible with an IT diagnosis,^3^ and this remains the consensus threshold for the MaGIC group. In the North American paediatric intergroup trial (INT-0106) of 44 patients aged 1–15 y, serum AFP was elevated in 34% of children with ovarian pure IT, with mean AFP level of 32 ng/ml (range 13–60 ng/ml). In IT with HL patients, 83% were observed to have elevated AFP, with mean levels of 304 ng/ml (range 80–1045 ng/ml).^20^ In a combined MaGIC analysis of paediatric and adult ovarian IT across four clinical trials, the mean AFP level was 83.0 ± 182.7 ng/ml in children and 63.2 ± 155.2 ng/ml in adults.^21^ In summary, whilst serum levels of AFP are typically higher in patients with IT with HL compared with those with pure IT alone, the relevance of these levels for determining optimal management, treatment. And prognosis remains unknown and is discussed in more detail in a later section.

The MaGIC expert consensus group opinion is that initial management is surgical, with further treatment dependent on the surgical findings. There are important differences in the approach to surgical staging between adult and paediatric surgeons. Tumours can be large at diagnosis, but importantly, tumour size does not influence staging or prognosis. The tumour-node-metastasis (TNM)^22^ and International Federation of Obstetrics and Gynecology (FIGO)^23^ staging systems are the most widely used for epithelial (e.g., carcinoma) and non-epithelial (e.g., GCT/IT) ovarian malignancies in adult patients. International guidelines for adult staging of epithelial cancer require intact removal of the affected ovary and ipsilateral fallopian tube with consideration of fertility-sparing surgery (preserving the contralateral ovary and fallopian tube, and the uterus) in selected patients. There is agreement that for patients of reproductive age with malignant OGCT, fertility-sparing surgery is appropriate. In addition, adult guidelines require a complete exploration of the abdomino-pelvic peritoneal cavity, peritoneal washings, peritoneal biopsies (pelvic peritoneum, paracolic gutters, diaphragm) and omentectomy (as least infracolic), along with removal of the pelvic and para-aortic lymph nodes up to the renal vessels bilaterally,^24^^,^^25^ although more recently European adult practice guidelines regarding such lymph node excision have been relaxed (see below).

The paediatric approach to surgical staging for patients with OGCT was modified in 2004 after a detailed review of the operative and pathology reports from the intergroup INT-0106 study.^26^ Although the intergroup protocol recommended use of the adult staging procedure, this was followed in only 3% of patients.^26^ It was found that biopsy of macroscopically normal appearing tissue was negative for tumour spread in all cases, and, furthermore, survival was excellent.^26^ From these results, a documentation-based surgical staging was recommended, including intact removal of the affected ovary with sparing of the ipsilateral fallopian tube if not macroscopically involved, with careful inspection and documentation of the contralateral ovary, diaphragmatic and peritoneal surfaces, pelvic and para-aortic lymph nodes and omentum, with biopsy only if abnormal appearances were found. Peritoneal fluid cytology should also be performed.^27^ Of note, the latest European guidelines for management of adolescents and adults with non-epithelial tumours now advocate lymph node excision only if there is evidence of nodal abnormality.^28^^,^^29^ These guidelines do, however, still recommend large omental biopsy even if normal in appearance, with removal recommended for cases with abnormal or adherent omentum.^28^^,^^29^ A report of 268 patients of all ages with OGCT, with apparent early stage disease and macroscopically normal omentum, showed only 3/268 (1.6%) patients had omental disease confirmed on histology.^30^ Of the 115 IT patients, there was no difference in disease-free (DFS) or overall survival (OS) relating to whether omentectomy was performed or not, regardless of age <18 y or ≥18 y.^30^

The reported incidence of positive lymph nodes in ovarian IT cases is up to 8%.^31^ However, the incidence of lymphoedema after pelvic and para-aortic lymphadenectomy is as high as 36% in some series.^32^ No impact on survival has been demonstrated in IT patients with apparent clinically early stage disease undergoing lymphadenectomy versus those who did not.^33^ Of 118 patients with IT (median age 24 y), 49% underwent lymphadenectomy and no survival difference was noted.^33^ The majority of these patients received adjuvant chemotherapy after surgery.^33^

Despite recommendations for comprehensive surgical staging during surgery for patients with OGCT, adherence to published guidelines is variable and staging compliance is not universally reported in published studies. Nonetheless, there is limited evidence to demonstrate that this affects patient outcomes. For example, a study of ovarian GCT patients, in whom 49 of 144 (34%) cases had IT, identified that incomplete surgical staging was associated with recurrence, as would be expected given the inclusion of malignant GCT subtypes such as dysgerminoma and YST.^34^ Of the 11 of 49 (22%) ovarian IT patients who relapsed, three were paediatric (<18 y), of whom two were not completely staged, with no deaths and eight were adults (≥18 y), of whom five were not completely staged, with one death reported.^34^ Thus, there was no association between relapse risk and extent of surgical staging in the IT cohort from this study. Consistent with this observation, in a study of 75 adult (≥18 y) stage I ovarian IT patients, complete staging surgery was not associated with DFS or OS, with excellent outcomes reported.^35^

To summarise this surgical staging section, the MaGIC group advocates that the minimum requirement for staging of all patients with ovarian tumours, whether paediatic or adult, should include 1) removal of the primary tumour, without violation of the tumour capsule if intact at the time of surgery (noting that spontaneous, non-iatrogenic rupture may already have occurred; if so this should be carefully documented); 2) inspection and biopsy of any abnormal-appearing peritoneal surfaces, lymph nodes, omentum, and contralateral ovary; and 3) peritoneal fluid cytology. Available evidence suggests that current adult protocols for comprehensive surgical staging risk removal of macroscopically-appearing normal structures with added morbidity and no obvious evidence of impact on survival. However, the extent of surgical staging in adults remains a potential area for further study, particularly with regards to excision of macroscopically normal lymph nodes. Data obtained from the AGCT0132 (ClinicalTrials.gov Identifier: NCT00053352) and AGCT1531 (NCT03067181) studies will provide further evidence with regards to staging using paediatiric and adult protocols.

Grade of ovarian IT (grade 1–3) was prognostic in the MaGIC US-UK analysis, with stage III, grade 3 patients being at ∼50% risk for recurrence compared with ∼10% risk for all other stage/group combinations.^21^ Management post-surgery typically differs between children and adults. In children, the standard-of-care has been surgery alone for ovarian IT,^3^^,^^21^ whereas in adult women, National Comprehensive Cancer Network (NCCN) and European Society for Medical Oncology (ESMO) guidelines recommend post-operative chemotherapy for all ovarian IT patients, except for those with appropriately-staged FIGO stage IA, grade 1 (low-grade) tumours. ESMO guidelines also acknowledge that for stage IA, grade 2 and 3, and stage IB-IC tumours, the need for adjuvant chemotherapy is controversial.^28^^,^^36^ There is no randomised trial comparing post-operative chemotherapy *versus* surveillance alone in this setting. However, in the MaGIC US-UK IT study, 90/98 paediatric patients were treated with surgery alone, whereas all 81 adult patients received post-operative chemotherapy. Despite differences in treatment, the outcome was similar even in patients with higher grade and stage disease.^21^ These data were recently corroborated by a MaGIC Brazilian IT study of 42 paediatric patients (n = 29 surgery alone; n = 13 received post-operative chemotherapy), with no difference in relapse risk by stage, grade, or chemotherapy delivery.^37^ Several recent studies in adults have questioned the role of post-operative chemotherapy.^38^, ^39^, ^40^ One study of 42 adult patients with ovarian IT showed that pre-operative chemotherapy delivery did not result in any radiological response, nor did chemotherapy pre-operatively or post-operatively reduce recurrence risk.^39^ Further, another study of 108 stage I (FIGO IA-C) adult ovarian IT patients (n = 81 surveillance; n = 27 post-operative chemotherapy) demonstrated no difference in relapse risk or OS between surveillance and chemotherapy patients; hence, surveillance alone for adults with FIGO IA-C disease was recommended.^40^ In addition, the stage I IT study of 75 adult patients described above also confirmed that delivery of adjuvant chemotherapy, and tumour grade, was not associated with DFS or OS.^35^ Together, these studies demonstrate the lack of chemotherapy response or outcome benefit for IT. Furthermore, platinum-based chemotherapy used for treatment of GCTs is associated with both short-term and long-term toxicity, the latter including cardiovascular disease, infertility, peripheral neuropathy, hearing impairment, and second malignancies.^41^ Together, based on all of these observations, our recommendation is close surveillance alone after surgery for all paediatric ovarian IT patients at initial presentation, regardless of stage or grade.^21^^,^^42^ This is because even for stage III, grade 3 patients, the relapse risk is ∼50%, and if relapse does occur, further surgery is the next recommended step.^21^ For adult ovarian IT patients, the published evidence supports surgery alone with surveillance for all stage I patients, regardless of tumour grade.^35^^,^^40^ Whilst the MaGIC expert consensus group believes it would also be reasonable to undertake surveillance for adult patients with higher stage (stage II-IV) disease, the group acknowledges that published evidence to support this statement is currently lacking and NCCN/ESMO guidelines still recommend adjuvant chemotherapy for these patient groups. Ideally, this question would be answered by a large, prospective international randomised controlled trial, where stage II-IV adult IT patients are randomised to surveillance alone versus adjuvant chemotherapy. We acknowledge that such a trial will be challenging to conduct due to the rarity of this entity.

Certain clinical scenarios deserve special mention, including management of ovarian IT patients with: presence of HL, elevated serum AFP, large volume ascites and/or pleural effusion, recurrence, and SMT, discussed in the following sections.

### Management of ovarian IT with microscopic foci of YST (Heifetz lesions; IT with HL)

The issue of how to classify and treat patients with ovarian IT with HL has been discussed in the recent MaGIC clinico-pathological conference report.^43^ Traditionally, only IT containing one or more foci of YST measuring >3 mm in diameter were classified as mixed GCT.^44^ However, as outlined in the 2020 WHO classification of female genital tumours, IT with any amount of YST, including microfoci of YST (IT with HL), is now defined as a mixed GCT.^2^ To facilitate management, pathologists recommend that any foci of YST be quantified, including size (diameter)—i.e., if ≤ 3 mm or >3 mm, and overall percentage of the tumour. Although IT with HL are now classified as mixed GCT histologically, from a clinical management standpoint, such patients are treated with surgery alone, particularly if completely resected. For patients with IT with HL that have residual disease post-surgery or for patients that present with advanced disease (> stage I), there is no consensus on the role of chemotherapy. Whilst most would advocate close clinical, radiological, and biochemical (serum AFP) monitoring, with consideration of chemotherapy for those with radiological recurrence and rising tumour markers, some may consider treatment upfront. That notwithstanding, the decision to now report patients with IT with HL as mixed GCT creates additional challenges. First, it represents a risk that inexperienced teams may over-treat such patients routinely by inappropriately delivering chemotherapy. Second, it represents a lost opportunity to study the outcomes for such IT with HL patients separately from both ‘pure’ IT (noting such ‘pure’ cases may well involve MT) and ‘true’ mixed GCT patients, if the amount of YST is not accurately quantified as highlighted above. This may hamper future study of optimal management for such patients and make it difficult to compare new trial data with old. Similarly, the decision to now grade IT patients merely as ‘low-grade’ and ‘high-grade’ (grades 2 and 3), also hampers the ability of the oncologist to appropriately and accurately counsel patients and families regarding recurrence risk for stage III disease [grade 1 and 2 patients; ∼10% recurrence; grade 3 patients ∼50% recurrence^21^]. As a further consequence, it will impede future studies of recurrence risk, as grading is now based on the less informative two-tier rather than previous three-tier system.

### Management of ovarian IT with elevated serum AFP

As discussed above, IT can present with elevated serum AFP. For fully resected, stage I ovarian IT patients (pure IT or IT with HL) with elevated serum AFP at initial presentation, the MaGIC expert consensus group recommends surveillance only for all patients (paediatric and adult), even with serum AFP >1000 kU/L at diagnosis, as would be routine for follow-up of e.g., a paediatric patient with fully resected stage I YST. For higher stage patients (e.g., those with residual disease following primary surgery and considered unresectable) with pure IT (including those with GP), controversy exists. Most in the MaGIC group, would continue to recommend a close surveillance approach for higher stage IT patients with AFP <1000 kU/L (noting that some might consider chemotherapy in this setting and that the NCCN/ESMO guidelines recommend chemotherapy for adult patients). For higher stage patients with elevated AFP >1000 kU/L, most in the MaGIC group would treat with chemotherapy (consistent with NCCN/ESMO guidelines for adult patients), however some would continue to recommend a close surveillance approach. Before consideration of a surveillance strategy in these higher stage cases with AFP >1000 kU/L, pathology review of the full resection specimen and discussion with clinicians with GCT expertise is advocated to ensure that mixed GCT elements have not been missed that are likely to alter management.

### Management of ovarian IT with large volume ascites and/or pleural effusion

In cases where the patient presents with massive ascites and/or pleural effusion, there are no published data to guide management. Pleural effusion in most patients is reactive and may be managed, if symptomatic, by pleurocentesis. Massive ascites may be a sign of widespread small volume peritoneal disease. Whereas in epithelial ovarian tumours, where such widespread small volume peritoneal disease with ascites would be a contraindication to upfront surgery, in IT, surgery remains the management of choice if the vast majority of disease is considered resectable, leaving only small volume residual. This is particularly pertinent as surgery becomes much more challenging if chemotherapy is delivered and GTS occurs (see below). Furthermore, the presence of ascites *per se* does not typically impact on the surgery itself, nor subsequent wound-healing. It may cause biochemical/renal instability post-operatively due to third space losses however, requiring close monitoring and replacement. If surgery is genuinely felt to be too high-risk in such patients, it may rarely be reasonable to consider 1–2 cycles of chemotherapy [e.g., ‘JEb’ (carboplatin, etoposide, and bleomycin once per cycle)^45^ or ‘PEb’ (cisplatin, etoposide, and bleomycin once per cycle)^46^], with surgical resection post-chemotherapy. It is also important in such rare cases where chemotherapy is to be considered to be confident that IT is the diagnosis from the overarching clinical, radiological, and biochemical picture. Where doubt exists, an ascitic tap for cytology and tissue biopsy (typically of the primary tumour) should be considered first. From our collective expert experience, response of the ascites/effusions to chemotherapy is variable in this scenario, and GTS may well occur. Accordingly, surgery remains the management of choice and use of chemotherapy must be very carefully considered with the above caveats, and cannot be routinely recommended in such cases.

### Management of ovarian IT at first recurrence or subsequent recurrences

Most patients with IT have an excellent outcome. In the MaGIC US-UK IT data analysis of paediatric and adult patients, event-free survival (EFS) and OS was 91% and 99%, respectively.^21^ A small subset of patients will recur and present with a new or enlarging mass, with or without a commensurate increase in serum AFP levels. Due to the paucity of cases, no studies have compared outcomes for surgery versus chemotherapy at relapse, but recent evidence of chemoresistance in IT^38^, ^39^, ^40^ adds weight to the argument for further surgery as first-line relapse management, particularly if a macroscopic (surgical and radiological) near complete resection can be achieved, leaving no more than minimal residual disease. The risks of resection need to be weighed against symptom burden, the rate of tumour growth, and the degree of compromise of surrounding vital structures. In cases where recurrence occurs on the contralateral ovary, fertility-sparing excision is advocated, with conservation of some ovarian tissue if at all possible.

Although complete surgical resection is ideal at recurrence, in cases where complete resection is not possible or would entail unacceptable morbidity, it may be preferable to resect large volume disease and observe minimally symptomatic, modest volume stable residual disease with close radiological and serial serum AFP estimation follow-up. This disease should not be ‘chased’ and cleared.^47^, ^48^, ^49^, ^50^ In such cases, more morbid surgery to remove residual disease can be reserved until the patient experiences symptoms or there is an imminent risk of compromise to a vital structure(s). In more widespread, irresectable recurrence, particularly in the very rare cases where there is parenchymal disease of the lung, liver or spleen, through presumed haematogenous spread or in patients with multiple recurrences where further resection is too morbid, there are no data to guide therapy. Although IT is chemo-resistant, there are certain clinical scenarios where it is reasonable to consider a trial of 1–2 cycles of chemotherapy with close surveillance. The reason for considering chemotherapy in these difficult scenarios stems from case reports showing that chemotherapy may be responsible for changing the behaviour and natural history of the IT, with e.g., low-grade IT and MT reported in the resected residual disease following chemotherapy, whereas high-grade IT was reported originally.^49^ Thus, in such cases chemotherapy is not delivered necessarily with the aim of reducing tumour volume or clearing it.^49^ This phenomenon has been called ‘retroconversion’ in the literature.^51^, ^52^, ^53^ There are no data to support one chemotherapy regimen over another in this setting. However, 1–2 cycles of JEb or PEb^45^^,^^54^ is reasonable to deliver in this scenario, after discussion with GCT experts.

Nevertheless, before considering chemotherapy in these select patients, it is important to counsel and have shared decision making with the patient and/or family, outlining the acute and potential long-term effects of chemotherapy, the potential that the tumour may not shrink radiologically, and indeed that the tumour may in fact appear to mature and grow, termed ‘growing teratoma syndrome’ (GTS).^19^ In such GTS cases, surgery has to be reconsidered and is likely to remain challenging (see below).

### Management of ovarian IT with growing teratoma syndrome (GTS)

There are rare instances in IT management where post-chemotherapy, there is enlargement of the residual mass, with normalised/normal serum tumour markers, termed GTS. At repeat surgery, pathology shows only MT, as first described in non-seminomatous GCTs,^19^ where there may have been treatment effect on the IT component. The aetiology of GTS is unknown, although biological studies are looking to explore this phenomenon in more detail.^55^ GTS is more common in males after chemotherapy for testicular GCT masses.^55^, ^56^, ^57^ In the literature, just over 100 cases of GTS have been described after an ovarian malignancy,^58^, ^59^, ^60^, ^61^ with incidence of 12–23% in ovarian IT. The aetiology and clinical behaviour of GTS in ovarian IT remains poorly understood by clinicians. In one study, the majority of these occurred after chemotherapy,^59^ whereas in a more recent report of 35 cases, 17 (49%) experienced GTS during chemotherapy and 18 (51%) after.^62^ Furthermore, there are reports of GTS occurring in ovarian IT patients with disease present without chemotherapy having even been delivered.^58^^,^^60^ Some factors have been reported which may predict the subsequent development of GTS, including residual disease, high-grade, and/or advanced stage IT disease, and the presence of GP at initial surgery.^62^^,^^63^

The importance of routine imaging cannot be underemphasised in the follow-up of ovarian IT patients, particularly those where the clinician has opted to treat with adjuvant chemotherapy rather than surveillance, and thus are at increased risk for the development of GTS, as most such recurrences are detected by routine radiological examination. Importantly, in a small series, some CT features have been described which occur alongside the development of GTS (i.e., when more MT compared with IT components are present), including increased density of mass lesions, with margins becoming better circumscribed in relation to adjacent tissues, and the onset of internal calcification, with fatty areas and cystic change.^52^

The primary management of GTS is surgical resection, and may require multiple surgical procedures. However, it should be noted that GTS often involves young patients with fertility desire. Therefore, fertility-sparing surgery should be performed in these GTS patients as spontaneous pregnancy has been reported.^60^^,^^62^ In addition, given GTS lesions are often extensive and may compromise multiple anatomical organs and structures, a multidisciplinary team (MDT) approach is advocated to consider management options to maximally reduce the bulk of the disease with the least morbidity. Patients with GTS should thus be transferred to high-volume specialised centres with access to multidisciplinary surgical care,^62^^,^^64^ as well as with expert GCT advice. However, in situations where complete surgical resection is not possible, uncontrollable local growth can result in substantial morbidity and even mortality. In these difficult situations, there is no established management approach. Interferon alpha, antiangiogenic agents, and cyclin (e.g., CDK4/6) inhibitors have been used with some reports of success,^65^, ^66^, ^67^, ^68^, ^69^, ^70^, ^71^, ^72^, ^73^, ^74^, ^75^ and can be considered although cannot be routinely recommended.

### Management of ovarian IT with somatic malignant transformation (SMT)

A subset of teratomas will undergo transformation to somatic malignancy (SMT), and this occurs most frequently, although not exclusively, after primary chemotherapy. Guidance for female patients should usually be informed from the more extensive experience of treating SMT in testicular cancer patients, albeit still a rare occurrence. In a series from China of adult women with ovarian IT, 2.5% of cases demonstrated SMT and patients were, on average, a decade older.^76^ There is evidence in both ovarian and testicular SMT that the transformed element is clonal, derived from the teratoma itself, essentially a manifestation of its pluripotency.^77^ One of the most frequent forms of SMT is into a neuroectodermal tumour, termed ENT.^16^ Historically, many patients with ENT were labelled as having PNET and assumed incorrectly to have Ewing sarcoma (of note, Ewing sarcoma can occur in the ovary but does so as a *de novo* tumour in the absence of teratoma).^16^ Other forms of SMT observed in teratoma include: carcinoma (adenocarcinoma or squamous cell carcinoma), neuroblastoma, and sarcoma (most frequently sarcoma not otherwise specified, but rarely rhabdomyosarcoma, leiomyosarcoma, etc). Surgical resection is the mainstay of therapy and it has been suggested that cure is unlikely in the absence of complete resection. Data from large international series of men with SMT suggests that if a patient is stage I (evidence of SMT in the primary gonadal tumour but no metastases), then surgery alone is sufficient and the prognosis remains favourable.^78^^,^^79^ For patients with evidence of SMT in metastatic sites, the choice of chemotherapy (if complete surgical resection is not possible) remains controversial. In the largest series of men with SMT, germ cell directed therapy was given to the majority of patients,^78^^,^^80^ whereas others have advocated for therapy directed against the transformed element.^81^^,^^82^ Eitherway, outcomes for patients with SMT present in metastatic sites remain poor.^78^^,^^80^, ^81^, ^82^ Consultation with experts is therefore advised on current management in these challenging cases.

### Biology of IT

The molecular characteristics that underpin the development of IT are poorly understood, due to a lack of study to date.^49^ It is known that 90% of tumours classified as grade 1–2 are diploid, whilst 66% of grade 3 tumours are aneuploid.^83^ Furthermore, grade 3 tumours most commonly harbour karyotypic abnormalities, likely to account for the increased propensity for recurrence in this group.^83^ Compared with other ovarian GCTs (such as dysgerminoma), ovarian IT show fewer DNA copy number changes.^83^ A recent multi-region exome sequencing study interrogated 52 distinct tumour components from ten females aged 8–29 years (median 17 years) with ovarian IT showed that these tumours were characterised by 2N near-diploid genomes, with extensive loss-of-heterozygosity (LOH) and an absence of recurrent somatic mutations or known oncogenic variants.^84^ Importantly, an identical pattern of LOH was seen across the genome, regardless of tumour component (IT, MT, or YST), indicating a shared clonal origin.^84^ This finding may have potential relevance for clinical management of patients compared with those presenting with other GCTs, such as pure YST. In contrast, bilateral ovarian IT cases showed distinct LOH patterns, consistent with independent clonal origins, and disseminated peritoneal disease, including GP, shared clonal LOH patterns with either the right or left primary ovarian IT.^84^ The neutral copy number LOH patterns observed indicate that diverse cell division errors during meiosis may contribute to ovarian IT pathogenesis.^84^ Finally, another area of unmet clinical need for patients with ovarian IT is a reliable biomarker. Serum AFP elevations may be observed in this disease and may well represent secretion from immature liver and gastrointestinal IT components, rather than true malignant YST elements.^85^ Circulating short, non-protein-coding RNAs termed microRNAs (specifically miR-371a-3p) are known to be elevated at malignant GCT diagnosis and negative for patients with MT.^86^ However, the role or otherwise of circulating microRNAs for patients with ovarian IT has not been studied to date. Collection of serum samples for such IT patients at diagnosis and into longitudinal follow-up on the prospective randomised clinical trial AGCT1531 will help to answer such questions. Accordingly, further molecular study of ovarian IT is required to improve our understanding of this rare disease and facilitate clinical management,^49^ and to that end, a recent article has highlighted such areas of both clinical and transational importance.^87^

## Conclusion

Ovarian IT is a rare and complex disorder and patients present to multiple clinical specialties across both paediatric and adult practice. Careful local multidisciplinary discussion is required in each and every case, both at initial presentation and again at any recurrence(s), to ensure considered and optimal management is delivered. Whilst surgery remains the mainstay of therapy, both at initial presentation and recurrence, there are some very select scenarios where consideration of 1–2 cycles of chemotherapy to assess response may be reasonable. Given the rarity of this entity, discussion with GCT experts, and/or expert national advisory panels, with relevant surgical, oncological, and pathological experience, where available, is recommended for such rare and complex patients.

## Outstanding questions

Further clinical and biological research is required to better understand and resolve current areas of controversy, and how some patients may optimally be managed, including identifying those who, for example, may safely undergo surveillance rather than chemotherapy strategies.

## Contributors

FP, MJM, JG, NM, JS, LK, TO, MH, ML, SS, ALF–Study conceptualisation.

FP, MJM, JG, NM, JS, DFB, LK, TO, MH, ML, SS, ALF–Literature review, data curation, interpretation, and analysis.

FP, MJM, JG, NM, JS, LK, TO, MH, ML, SS, ALF–Writing–original manuscript draft.

FP, MJM, JG, NM, JS, DFB, LK, TO, MH, ML, SS, ALF–Writing–review and editing, approval of final manuscript, including revisions. All authors read and approved the final version of the manuscript.

## Declaration of interests

The authors have no conflicts to declare.
